# Dual-Source Linear Energy Prediction (LINE-P) Model in the Context of WSNs

**DOI:** 10.3390/s17071666

**Published:** 2017-07-20

**Authors:** Faisal Ahmed, Gert Tamberg, Yannick Le Moullec, Paul Annus

**Affiliations:** 1 Thomas Johann Seebeck Department of Electronic, Tallinn University of Technology, Tallinn 12616, Estonia; paul.annus@ttu.ee; 2 Department of Cybernetics, Tallinn University of Technology, Tallinn 12616, Estonia

**Keywords:** WSN, energy harvesting, transient computing

## Abstract

Energy harvesting technologies such as miniature power solar panels and micro wind turbines are increasingly used to help power wireless sensor network nodes. However, a major drawback of energy harvesting is its varying and intermittent characteristic, which can negatively affect the quality of service. This calls for careful design and operation of the nodes, possibly by means of, e.g., dynamic duty cycling and/or dynamic frequency and voltage scaling. In this context, various energy prediction models have been proposed in the literature; however, they are typically compute-intensive or only suitable for a single type of energy source. In this paper, we propose Linear Energy Prediction “LINE-P”, a lightweight, yet relatively accurate model based on approximation and sampling theory; LINE-P is suitable for dual-source energy harvesting. Simulations and comparisons against existing similar models have been conducted with low and medium resolutions (i.e., 60 and 22 min intervals/24 h) for the solar energy source (low variations) and with high resolutions (15 min intervals/24 h) for the wind energy source. The results show that the accuracy of the solar-based and wind-based predictions is up to approximately 98% and 96%, respectively, while requiring a lower complexity and memory than the other models. For the cases where LINE-P’s accuracy is lower than that of other approaches, it still has the advantage of lower computing requirements, making it more suitable for embedded implementation, e.g., in wireless sensor network coordinator nodes or gateways.

## 1. Introduction

Although improvements have been made in the domain of energy storage (e.g., supercapacitor and lithium battery), those storage devices still have numerous shortcomings such as size, installation, maintenance and cost, especially for WSNs nodes [1]. In this context, energy harvesting is an increasingly popular approach used for powering wireless sensor network (WSN) nodes. Various energy harvesting methods and techniques have been proposed and developed over the last decade [2]; such approaches are typically used to complement more traditional energy storage devices such as rechargeable batteries and supercapacitors, or even to replace them all together as in battery-less nodes that operate according to the principles of transient computing [3].

However, energy sources such as solar and wind are characterized by significant variations and intermittence; thus, it is challenging to guarantee that the WSN nodes always have the necessary energy to operate. In turn, this can negatively impact the quality of service of the application. In the worst case, some nodes might temporarily run out of energy. To alleviate this issue, on-line mechanisms such as dynamic duty cycling and/or dynamic frequency and voltage scaling can be used to modulate the energy consumption of the WSN node according to the available energy. 

In this context, energy prediction plays a vital role to deal with questions such as “when is the next power loss going to happen?” and “What will happen when the data transmission/reception is performed although energy has not been predicted properly?” In addition, “Will WSNs operation provides satisfactory QoS by using energy prediction, specifically if used together with transient computing?” The importance of energy prediction in WSNs is also highlighted by recent works such as [4] which proposes a forecast algorithm applicable to solar-powered WSNs and also demonstrates its practical implementation using real WSN nodes, as well as [5] which focuses on a wind energy prediction model. In particular, the accuracy of the prediction or forecast model is deemed significant, especially in the case of autonomous WSNs for which proper operation relies on the available energy predictions [4].

Moreover, research shows that, at least as of today, a node’s radio chip consumes the largest amount of energy as compared to computation and sensing operations [3]. Proper management of radio consumption can be more effective if the microcontroller of a WSN node can be programmed in such a way that it performs the transmission/reception operations in accordance to the predicted energy availability. 

Furthermore, other causes of energy wastage in the WSN nodes are idling, listening, etc. As a solution, a node remains in the idle state and wakes up when the energy is predicted and performs transmission/receiving at that time. Energy prediction can be seen as an alternative solution [3], which can control the computation and communication operations in the WSN nodes, although energy optimizations techniques can also be applied with modifications, e.g., in the MAC (media access control) protocol [6].

For better performance of the autonomous WSN nodes, energy prediction concept is essential because prediction at different data time intervals provides more accuracy, realistic results, and allows executing the tasks when energy has been properly estimated [7].

Although it could be argued that energy prediction in the context of energy harvesting technologies for WSNs is now quite mature, few energy prediction models provide accurate results at a low computational complexity cost. In fact, energy prediction for autonomous WSNs is still not extensively explored, which calls for further research. Most of the prediction models use as much as possible the energy history (e.g., past records) for accuracy [7] or by employing rather computational complex models to reduce the error estimation. On the contrary, in this article, we propose three different sub-cases of an energy prediction model, named LINE-P, which considers very few values for predicting energy from past records. Furthermore, most proposed energy prediction models are suitable for solar energy or wind energy only; to accommodate the emergence of multi-source energy harvesting, the proposed LINE-P model supports dual-source (solar and wind) WSNs harvesters.

The main contributions presented in this paper are:

1. An overview of existing fixed weighting factor based energy prediction models.
2. A proposal for a symmetrical kernel-based model (LINE-P) for dual-source (solar and wind) which estimates the value on three different data time intervals, i.e., shorter, medium and longer. Indeed, although different prediction models have been proposed in the literature to forecast solar or wind energy availability, most are based on a fixed weighting factor. However, the fixed weighting factor is incompatible with the solar powered WSNs because each solar panel has a different set of parameters [4]. On the other hand, the symmetric kernels have simple computation of the dot product in a potentially infinite dimensional feature space by means on the kernel function. In addition, symmetric kernels have a simpler structure than non-symmetric kernels.
3. A comparison of the proposed LINE-P model against state-of-the-art energy prediction models (fixed weighting factor) for solar and wind-based energy sources. We validate our model by using real datasets (energy profiles) and comparing the performance of the various models by means of classical error estimation techniques, showing their accuracy and complexity in terms of execution time and space (memory).

Before looking at the details of the existing and proposed models, what follows briefly discusses solar and wind energy; these two sources as used for the experimental results described in the second part of this paper. 

### 1.1. Solar Energy 

In consumer applications, the concept of solar energy harvester came up in the late 1980s [8], as illustrated by many applications such as calculators and electronic games that were powered by means of solar harvested technology. Solar energy harvesting converts light and heat from the sun into electricity. Nevertheless, the direction of the solar panel is very crucial; i.e., two co-located harvesters at different angles produce different amounts of energy. In addition, indoor solar energy harvesting is generally speaking less exploited as it generates less energy [8].

Both outdoor and indoor solar energy harvesting can potentially power a system for relatively long durations, although due to their uncertainties (either varying weather or varying indoor illumination patterns), neither can be used very in a dependable way, especially when considering autonomous and transient computing based nodes.

### 1.2. Wind Energy

Nature provides us many non-polluting energy sources, including wind. Three key elements affect the amount of energy that can be harvested from wind, i.e., wind speed, air density and shaft area. A small change in these elements causes large differences in the net amount of energy, either positively or negatively. It has also been shown that no wind turbine converts more than 59.3% of the kinetic energy of the wind into mechanical energy [8].

### 1.3. Datasets

In order to design the proposed energy prediction model, as well as to evaluate and compare its performance, several datasets have been used. Significantly, solar and wind technology are varying and intermittent by nature. For solar energy, we considered two different data time intervals, shorter and medium; since wind energy is very uneven, a longer data time interval is better for improving the prediction accuracy. However, a longer time interval (more number of slots) requires more space.

To fulfill the different data time interval requirements, we obtained datasets from trusted sources for different locations.

From the California ISO (Folsom, CA, USA), we selected three datasets for solar energy, Southern California Edison Company (SCE, Rosemead, CA, USA), Pacific Gas and Electric Company (PG&E, San Francisco, CA, USA), San Diego Gas & Electric Company (SDG&E, Santa Ana, CA, USA), and one dataset for wind energy [9].

Shorter, medium and longer data time intervals of 22, 60 and 15 min, consisting of 24, 61 and 96 slots in 24 h, respectively, have been used. Furthermore, data from NREL’s Solar Radiation Research Laboratory (SRRL, Washington, DC, USA) [10] were used for one solar energy profile (shorter data time interval). 

Finally, we used one profile for wind energy from Elia (Belgium’s electricity transmission system operator) [11] specifically for longer data time interval.

## 2. Materials and Methods

In the context of WSNs, few prediction models for solar and very few for wind energy exist. This section comprises two parts: in the first one, the state of the art related to solar and wind based energy prediction models is discussed in detail; and, in the second part, we describe and discuss the proposed LINE-P model.

### 2.1. Solar-Based Energy Prediction Models

Solar energy is considered on short-term intervals for accurate prediction purposes, i.e., a day is divided into slots ranging from one minute to several hours [12]. For example, in [12], a day is divided into 24 slots (an hour equal to one slot).

#### 2.1.1. Exponential Weighted Moving Average (EWMA)

EWMA [13] is one of the popular prediction models in the domain of WSNs. Several models have then been proposed to extend EWMA [6,13,14]. EWMA predicts the solar energy based on the energy profile of the previous day along with the historical average of real data [15]. EWMA has been discussed in [6] and is expressed as:

(1) X(i)=αX(i−1)+(1−α)x(i)

where *x*(*i*) denotes the value of the real energy. EWMA is dependent on the weighting factor α, which ranges from 0 to 1, and *x*(*i*), which expresses the real energy. EWMA works very well on longer slots and if the weather is consistent. However, EWMA is not suitable for shorter slots and generates large errors for alternate sunny and cloudy days [15].

**Complexity of EWMA**: In this work, we are interested in comparing the complexity (in terms of running time) of various energy estimation methods. For this, we present the Big-O notation for each of them, starting with that of EWMA for a single estimation value. Since in Equation (1) the number of multiplication operations are constant and one addition operation is performed, the complexity in terms of running time is denoted by *T*(*n*); i.e., *T*(*n*) = 2, thus the Big-O for EWMA is *O*(2).

#### 2.1.2. Weather Conditioned Moving Average (WCMA)

WCMA is an extension of EWMA that works on short-term prediction by accounting for the mean of the previous day’s energy as well as the mean of the current day’s energy [14]. WCMA is proposed in [6] and expressed as:

(2) $$E\left(d,n+1\right)=\alpha E\left(d,n\right)+GAP_{K}\left(1-\alpha \right)M_{D}\left(d,n+1\right)$$

The estimation yielded by WCMA is more accurate and has a lower computational complexity as compared to EWMA [16]. In Equation (2), α is a weighting factor similar to that used in Equation (1), E(d,n) is the harvested energy of the previous slot, $M_{D}\left(d,n+1\right)$ is the mean of the D past days at *n* + 1 sample of the day, and $GAP_{K}$ is a new factor which reflects the solar condition in the present day on the base of the previous day [15], and E(d,n+1) is represent predicted energy for the next slot. In [15], the authors presented a comparative analysis of EWMA with WCMA and found higher accuracy for WCMA based on four different day profiles. Considering *K* = 3 and α = 0.7 for both models, the mean square error (MSE) and mean absolute error (MSA) of WCMA is less than that of EWMA, i.e., 5% and 7%, respectively.

**Complexity of WCMA:** WCMA introduces the $GAP_{K}$ factor that depends on the present and previous days, so $GAP_{K}$ complexity in terms of running time is *T*(*n*) = *n*^2^ (*k* + 1), where *n* is the length of the vector and *k* is the number of previous days. $M_{D}\left(d,n+1\right)$ is the mean of the past days, so it is *T*(*n*) = *nk*. The total complexity of WCMA in terms of running time is *T*(*n*) = *k* (*n*^2^ + 1) and with the Big-O notation it is O(*n*^2^), whereas the other parameters are negligible.

#### 2.1.3. Accurate Solar Energy Allocation (ASEA)

ASEA is also based on EWMA. In [16], the author realized the importance of short-term conditions, and designed the ASEA model keeping in mind situations where the weather is extremely unpredictable such as in the northern part of Europe, etc. To address the above problem, the authors of ASEA introduced the parameter ψ as a weighting factor. It is based on the ratio between the harvested energy and real energy data, based on the previous slots. For example, when ψ is smaller than 1, it indicates bad weather or other issues.

The ASEA model is expressed as:

(3) $$E\left(d,n\right)=E\left(d,n\right).\psi ;\;where\;\psi =\frac{H\left(d,n-1\right)}{E\left(d,n-1\right)}$$

where *E* is the predicted value of EWMA and *H* is the harvested energy.

**Figure 1:**
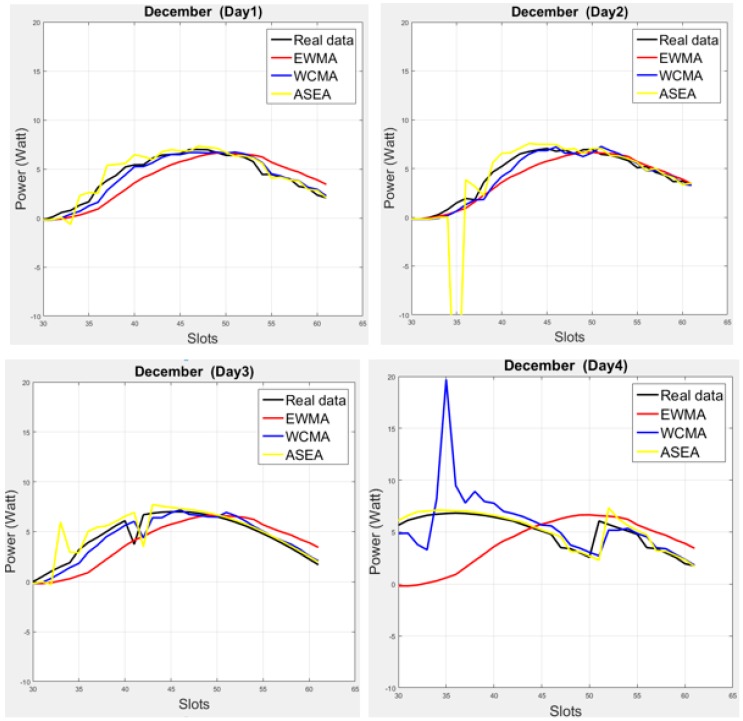
Graphical comparison of three energy prediction models (EWMA, WCMA, and ASEA) with real solar energy data for four different days in December. While the three models can follow the general trend of the real data, none of them can deal with all illumination variations due to inconsistent weather conditions.

Moreover, ψ is calculated at the start of each slots, and then multiplied with EWMA for the ASEA prediction values. In [17] the authors have checked the performance of ASEA on the summer season; usually the weather was consistent at that time. However, we have verified the performance of ASEA by utilizing three different data profiles (for three different months, i.e., in August, October and December) and we found that ASEA is not always closer to real data than WCMA, as Figure 1 illustrates for the month of December on the dataset presented in [9].

**Complexity of ASEA:** ASEA introduces the ψ factor which has *T*(*n*) = 1. This is then multiplied with the value of EWMA as can be seen in Equation (3). Thus, the total complexity of ASEA in terms of execution time is *T*(*n*) = 2. The Big-O notation of ASEA is *O*(2).

#### 2.1.4. A Solar Energy Algorithm with Q-Learning (QL-SEP)

QL-SEP is a solar energy prediction model that has been recently proposed in [12]. It uses the historical data of past days and as well as most recent weather condition from the present day. In [12], the author assumes that solar energy is based on a periodic cycle and they thus equally divide each day into many slots. QL-SEP also uses the feature of EWMA for the current solar condition. Furthermore, the author introduces a daily ratio (DR) parameter. DR is the average of the energy either increasing or decreasing in the previous slots. DR can be computed as:

(4) $$DR=\frac{\sum_{i=1}^{N}\left(P_{e}\left(i\right).R\left(i\right).i\right)}{\sum i}.$$

In Equation (4), $P_{e}$ expresses the prediction error, R is the reliability level and i is the index. R is the key factor which represents the current reward (status) [12]. Suppose the harvested energy *H* is that of the prediction energy of EWMA as shown in Expression (5):

(5) $$\frac{\left|H-P\right|}{P}$$

Therefore, if the result of Equation (5) is positive, then R is considered as +1, otherwise −1, when calculating R for each slot. In addition, the value of R changes the status of *r* as per γ which is the learning rate with the value of 0.1 in [12]. These parameters are applied in Equation (6) to calculate the Q-value:

(6) $$Q_{t+1}\left(s\right)=Q_{t}\left(s\right)+\gamma \left(r-Q_{t}\left(s\right)\right)$$

After calculating the Q-values, DR is obtained as:

(7) $$DR=\frac{\sum_{i=1}^{N}\left(\frac{\left|H-P\right|}{P}\right).Q\left(i\right).i}{\sum \text{​}i}$$

Finally, the QL-SEP predicts the energy based on DR and EWMA, as expressed in Equation (8):

(8) $$E_{\text{QL-SEP}}=E_{\mathrm{EWMA}}\left(1+DR\right)$$

In [12], the author evaluates the QL-SEP models on real-life solar data over a one-year period and achieves better estimation comparatively to EWMA, ASEA, and Pro-Energy. However, QL-SEP is designed for longer slots; for instance, each day is divided into 24 slots [12], which is not suitable if the weather changes rapidly and continuously; furthermore, to get accurate results, a significant number of computations are required since the device running the prediction modeling has to perform the calculations for EWMA and then for QL-SEP.

**Complexity of QL-SEP:** In Equation (8), DR is dependent on the Q-value, as can be seen in Equation (7), so the complexity in terms of running time of DR is *T*(*n*) = (2*n* + 1)*q*. Then, for obtaining the final value of QL-SEP, this is multiplied with EWMA. Now, *T*(*n*) = (4*n* + 2)*q*, and the Big-O notation of QL-SEP is *O*(*n*); the other parameters are negligible.

#### 2.1.5. Pro-Energy Prediction Model (Suitable for Solar and Wind)

Pro-Energy (PROfile Energy prediction model) predicts energy based on the past days [12]. The Pro-Energy model is designed for multi-source (solar and wind) and is recommended for short and medium slots in a given day. Pro-energy matches the information of the current day with the most similar day among the pool of stored energy profiles. In addition, Pro-energy predicts the next value with the combination of the next slot in the stored profile noted in the last slot [14].

The energy for the current day is calculated as:

(9) $$E\left(d,n\right)=\alpha H+\left(1-\alpha \right)E_{\mathrm{MS}}$$

where *H* is the harvested energy in the previous slot and $E_{\mathrm{MS}}$ is the observed energy for the most similar day. For evaluating the similarity between the previous day and the current day, the mean absolute error (*MAE*) of each day from previous to current slot is calculated and stored in *K*. The smallest *MAE* of any day is considered as the most similar day [14]. For multiple profiles, $E_{\mathrm{MS}}$ is replaced with a weighted profile (*WP*) that is computed as:

(10) $$WP=\frac{\sum_{j=0}^{P}w_{j}.E_{sj}}{P-1}$$

Pro-energy combines multiple energy profiles in order to get better estimates for different data time intervals. In Equation (10), P represents the profiles, *MAE* of each day is stored in *E*, and $w_{j}$ is calculated as:

(11) $$w_{j}=1-\frac{MAE\;(E_{sj},C)}{\sum_{j=1}^{P}MAE\;(E_{sj},C)}$$

where *C* is the current day. By inserting Equation (11) into Equation (10), and for multiple profiles, Equation (9) becomes Equation (12), i.e., the energy prediction model of Pro-Energy:

(12) E(d,n)=αH+(1−α)WP

In [14], the authors evaluate the performance of Pro-energy by deploying TelosB nodes with Solar PV and wind micro-turbines energy harvesters along with datasets from the US National Renewable Energy Laboratory. Their results show 60% better prediction than EWMA and WCMA. 

**Complexity of Pro-Energy:** The Pro-Energy model expressed in Equation (12) is based on multiple profiles and requires a significant number of computations. *K* stores the mean absolute error (*MAE*) of previous and current slots, so its complexity in terms of running time in relation to $w_{j}$ is *T*(*n*) = (*k* + 1)^2^ and that of WP is *T*(*n*) = *n*. Overall, the running time complexity of Pro-energy is *T*(*n*) = (*k* + 1)^2^*n*, i.e., higher than that of the previously analyzed models because of the squaring factor. The Big-O notation of the Pro-Energy model is *O*((*k* + 1)^2^).

## 3. Proposed Dual-Source (Solar and Wind) Linear Energy Prediction) Model (LINE-P) 

In this section, we discuss the proposed linear energy prediction model, of which the aim is to reduce the computational complexity while maintaining similar accuracy as compared to the other models. 

In order to predict the amount of the harvested energy in the next time slot, we propose a class of methods based on sampling operators. We suppose that the energy profile *E* can be expressed as:

(13) $$E\left(t\right)=E^{*}\left(t\right)+\vec{E}\left(t\right)$$

where $E^{*}$ is a smooth trend and $\vec{E}$ represents fluctuations. Our aim is to construct a predictor that on the one hand is good for approximation of smooth trends expressed by $E^{*}$ and, on the other hand, is not so sensitive to fluctuations expressed by $\vec{E}$. In our approach, we use results of approximation and sampling theory. In the following, we provide a short overview of those results.

### 3.1. Sampling Operators

For the uniformly continuous and bounded f ∈ C(ℝ), the generalized sampling series are given by (t ∈ ℝ;w>0) as Equation (14), i.e., a summation of function values with sampling kernel,

(14) $$\left(S_{w}f\right)\left(t\right)≔\overset{\infty }{\underset{k=-\infty }{\sum }}f\left(\frac{k}{w}\right)s\left(wt-k\right),$$

where s ∈ C(ℝ) is a kernel function (see Definition 1 below).

If the kernel function, used in sampling series is the cardinal sine or sinc function, as:

$$s\left(t\right)=sinc\left(t\right)≔\frac{\sin\pi t}{\pi t},$$

we get the classical Whittaker-Kotel’nikov-Shannon sampling operator:

(15) $$\left(s_{\omega }^{sinc}f\right)\left(t\right)≔\overset{\infty }{\underset{k=-\infty }{\sum }}f\left(\frac{k}{w}\right)sinc\left(wt-k\right),$$

Let us take w=1 and t=j ∈ ℤ in Equation (14), then

(16) $$\left(S_{1}f\right)\left(j\right)≔\overset{\infty }{\underset{k=-\infty }{\sum }}f\left(k\right)s\left(j-k\right),$$

The idea to replace the sinc kernel $sinc\left(\cdot \right)\notin L^{1}\left(\text{ℝ}\right)$ by another kernel function $s\in L^{1}\left(\text{ℝ}\right)$ appeared first in [18], where the case $s\left(t\right)={\left(sinc\left(t\right)\right)}^{2}$ was considered. A systematic study of sampling operators (14) for arbitrary kernel functions was initiated in 1977 at the RWTH Aachen University by Butzer and his students [19,20,21].

In [20], Section 4 describes why we should be motivated to use the generalized sampling operators (Equation (14)) and also describes the general convergence theorems and convergence theorems with rates.

### 3.2. Kernels 

The general kernel for the sampling operators (Equation (14)) is defined in the following way. Definition 1 [20] if s:ℝ→ℂ is a bounded function such that:

(17) $$m_{0}\left(s\right)≔\overset{\infty }{\underset{k=-\infty }{\sum }}\left|s\left(u-k\right)\right|<\;\infty \;\left(u\in \text{ℝ}\right),$$

with the absolute convergence uniform on compact subsets of ℝ, and

(18) $$\overset{\infty }{\underset{k=-\infty }{\sum }}s\left(u-k\right)=1\;\left(u\in \text{ℝ}\right),$$

Now, s is said to be a kernel for sampling operators (14).

The objective of this paper is to use results from [22,23] for signal prediction with the generalized sampling operators ((Equation (14)), when the kernel function s is defined via the Fourier transform of certain even window function $\lambda \in C_{\left[-1,1\right]},\;\lambda \left(0\right)=1,\;\lambda \left(u\right)=0\;\left(\left|u\right|\right)\geq 1.$ More precisely, our kernel function is defined by the Equation (19),

(19) $$s\left(t\right)≔s\;\left(\lambda ;t\right)≔\int_{0}^{1}\;\lambda \left(u\right)\cos\left(\pi tu\right)du.$$

This approach generates even kernels. For some cases, asymmetric kernels are more appropriate. In this case, we use a general window function λ:[−1,1]→ℂ and define the kernel in the Equation (20),

(20) $$s\left(t\right)≔s\;\left(\lambda ;t\right)≔\frac{1}{2}\int_{-1}^{1}\;\lambda \left(u\right)\exp\left(-i\pi tu\right)du.$$

In [24], we considered the general cosine window:

(21) $$\lambda_{C,a}\left(u\right)≔\overset{n}{\underset{k=0}{\sum }}a_{k}\cos k\pi u\;(n\in \text{ℕ},\;a=\left(a_{0},a_{1},\ldots ,a_{n})\right),$$

provided:

(22) $$\overset{\left\lfloor \frac{n}{2}\right\rfloor }{\underset{k=0}{\sum }}a_{2k}=\;\overset{\left\lfloor \frac{n+1}{2}\right\rfloor }{\underset{k=0}{\sum }}a_{2k-1}=\;\frac{1}{2}.$$

We get the Hann window, if we take n=1 in (21) and Blackman window, if *n* = 2 and $a_{0}=a$ in Equation (21). For n∈ℕ, there exists a choice of parameters, which allows us to have the order of approximation of the corresponding sampling operators estimated by $\omega_{2n}\left(f;\frac{1}{w}\right)x$ [19]. Another choice of the parameter vector $a=a^{*}$ in Equation (21), where the parameter vector $a\in {\text{ℝ}}^{n+1}$ has components $a_{0}^{*}=\frac{1}{2^{2n}}\;\left(\begin{array}{c} 2n\\ n \end{array}\right)$ and $a_{k}^{*}=\frac{1}{2^{2n-1}}\;\left(\begin{array}{c} 2n\\ n-k \end{array}\right)$ for k=1,2,…,n, gives us by Equation (19) a family of rapidly decreasing kernels $s_{H,2n}=O(|t{|}^{2n+1})$ (see [24] for corresponding operator norms and [25,26,27] for truncation errors).

The general cosine window generates a linear combination of translated sinc-functions. We can use instead of the general cosine window a window in the Equation (23),

(23) $$\lambda_{E,a}\left(u\right)≔\overset{n}{\underset{k=-n}{\sum }}a_{k}e^{ik\pi u}\;(n\in \text{ℕ},\;a=\left(a_{-n},a_{-n+1},\ldots ,a_{n})\right)\in {\text{ℝ}}^{2n+1},$$

provided:

(24) $$\overset{\left\lfloor \frac{n}{2}\right\rfloor }{\underset{k=-\lfloor \frac{n}{2}\rfloor }{\sum }}a_{2k}=\;\overset{\left\lfloor \frac{n+1}{2}\right\rfloor }{\underset{k=1-\lfloor \frac{n+1}{2}\rfloor }{\sum }}a_{2k-1}=\;\frac{1}{2}.$$

If we use Equation (20), we get a corresponding kernel in the Equation (25),

(25) $$s_{E,a}\left(t\right)=\overset{n}{\underset{k=-n}{\sum }}a_{k}\mathrm{sinc}\left(t-k\right),$$

which is indeed a kernel in terms of Definition 1, because Condition (24) guarantees that we have Equation (18) and that $m_{0}(s_{E,a})$ is bounded. Let w=1 and t=j ∈ ℤ in Equation (14), then for a kernel $s_{E,a}$ we get

(26) $$(S_{1;E,a}f)\left(j\right)≔\overset{\infty }{\underset{k=-\infty }{\sum }}f\left(k\right)s_{E,a}\left(j-k\right)=\overset{n}{\underset{k=-n}{\sum }}a_{k}f\left(t-k\right).$$

#### 3.2.1. Approximation Error Estimates

We estimate the approximation error in terms of modulus of smoothness. The classical modulus of smoothness ([28], p. 76) is defined for any δ>0 by

$$\omega_{k}\left(f;\delta \right)c≔\underset{\left|h\right|\leq \delta }{\sup}||\Delta_{h}^{k}f\left(\cdot \right)||C,\;f\in C\left(\text{ℝ}\right),$$

where the one-side difference in respect to increment h is given by:

(27) $$\Delta_{h}^{k}f\left(x\right)=\overset{k}{\underset{l=0}{\sum }}{\left(-1\right)}^{k-l}\left(\begin{array}{c} k\\ l \end{array}\right)f\left(x+lh\right).$$

Modulus of smoothness is a neat measure of the structural properties of a function. As we can see from the definition, the modulus of smoothness is related to the derivative of the function. We can estimate the *r*-th modulus of smoothness using the *r*-th derivative of a function. Our aim is to construct a predictor in the form of a sampling operator that has approximation error estimate via modulus of smoothness of high order. Such predictors are good for the approximation of smooth trends (i.e., trends with high order continuous derivatives).

We proved in [23] a theorem about the approximation properties of the Blackman–Harris sampling operators, defined by the general cosine window (Equation (21)).

#### 3.2.2. Theorem 1 [23]

For $C_{w,a}\left(a\in {\text{ℝ}}^{n+1}\right)$, let l,1≤l≤n be fixed. If *l* = 1 or for every *j* = 1,…,*l* − 1

$$\sum_{k=1}^{n}a_{k}k^{2j}=0,$$

then, for f∈C(ℝ), we have estimated order of the approximation,

$$||C_{w,a}f-f||\leq M_{a,l}\omega_{2l}\left(f;\frac{1}{w}\right).$$

Then, constant *M_a,l_* is independent of *f* and *w*.

For the sampling operators, defined by the general exponent window in Equation (23), we need to prove an analogous theorem. Because we need to use samples from the past to predict the current value, we give a theorem for one-sided kernels, i.e., we use a parameter vector *a* such that $a_{k}=0\;\mathrm{for}\;k\leq 0.$

#### 3.2.3. Theorem 2 [23]

For $S_{w;E,a}$ with $\left(a\in {\text{ℝ}}^{n+1}\right)$ such that $a_{k}=0$ for k≤0, let l,1≤l≤n be fixed. 

If *l* = 1 or for every *j* = 1,…,*l* − 1

(28) $$\sum_{k=1}^{n}a_{k}k^{2j}=0,$$

and for every *j* = 1,…,*l*

(29) $$\overset{k}{\underset{k=1}{\sum }}{\left(-1\right)}^{k}a_{k}k^{j}=0,$$

then, for f∈C(ℝ), we have estimated order of the approximation,

(30) $$||S_{w;E,a}f-f||\leq M_{a,l}\omega_{l}\left(f;\frac{1}{w}\right).$$

Then, constant *M_a,l_* is independent of *f* and *w*. In [21], Theorem 2 has been proven.

#### 3.2.4. Good Kernels for Prediction

Theorem 3 [28]

Let s ∈ C(ℝ) be a kernel. Then, ${\left\{s_{w}\right\}}_{w>0}$ defines a family of bounded linear operators from C(ℝ) into itself with the operator norm $||S_{w}\left|\left|\equiv ||S_{w}\right|\right|c\to c$, satisfying

$$||S_{w}||=\underset{u\in \text{ℝ}}{\sup}\sum_{k=-\infty }^{\infty }\left|s\left(u-k\right)\right|<\infty \;\left(w>0\right).$$

If we suppose that the energy profile E can be represented in form

$$E\left(t\right)=E^{*}\left(t\right)+\vec{E}\left(t\right),$$

where $E^{*}$ is a smooth trend and $\vec{E}$ represents fluctuations, then we have

$$(S_{w}E)\left(t\right)=(S_{w}E^{*})\left(t\right)+(S_{w}\vec{E})\left(t\right),$$

and the error of predicting the trend is

$$|(S_{w}E)\left(t\right)-(S_{w}E^{*})\left(t\right)\left|=|(S_{w}\vec{E}\left(t\right)\left|\;\leq \underset{t}{\sup}\;\right|\vec{E}\left(t\right)\left|\;\right|\left|S_{w}\right|\right|.$$

The last estimate indicates that for good prediction we need to choose a sampling operator with a small norm. If the trend is smooth [18], we need for good approximation a kernel with approximation error estimate via high order of approximation.

We choose a symmetric kernel (Equation (25)) with the parameter vector

$$b≔\left\{-\frac{11}{2560},-\frac{11}{256},-\frac{23}{320},\frac{25}{256},\frac{167}{512},\frac{25}{128},0,\frac{25}{128},\frac{167}{512},\frac{25}{256},-\frac{23}{320},-\frac{11}{256},-\frac{11}{2560}\right\}$$

The symmetric kernels have simpler computation and structure than non-symmetric kernels.

For this b-kernel, we have Theorem 1, and, for *a*-kernel, we use Theorem 2, which provides estimates of the error of approximation via modulus of smoothness order 4. This kernel also has a good decay and a small operator norm, close to the minimal possible value of the norm for a kernel with such order of approximation.

We choose a one-sided kernel (Equation (25)) with the parameter vector

$$a≔\left\{0,0,0,0,0,0,0,\frac{3}{8},\frac{15}{16},\frac{1}{2},-\frac{3}{8},-\frac{3}{8},-\frac{1}{16}\right\}$$

In the following, we construct predictors as sampling operators in Equation (26) with kernels using those parameter vectors.

## 4. Prediction

We define three predictors using sampling operators in Equation (26). For the first case, we use the previous samples from the same day and the information from one of the previous days, closest to the current day. Because the symmetric kernels give better order of approximation, we use in our predictor a symmetric kernel with parameter vector *b*. For measure of the closeness and error correction, we use a one-sided kernel with parameter vector *a*.

For the second case, we use only the previous samples from the same day and a one-sided kernel. The third case is a simplified version of the first case. Instead of the one-sided kernel, we use a part of the main symmetric kernel for measure of the closeness and error correction.

### 4.1. Case-I

If we have the samples $f_{l}\left(l=1,\ldots ,k\right)$ from k previous days, then we can use this information for more complex prediction method. The parameter vector *b* defines a symmetric kernel, the parameter vector *a*, where $a_{k}=0$ for k≤0, generates a one-sided kernel with the corresponding sampling operator (Equation (26)), yielding Equation (31),

(31) $$(S_{PREDI;b}f)\left(j\right)≔\sum_{k=1}^{m}b_{k}f\left(j-k\right)+\sum_{k=-m}^{0}b_{k}\;f_{l}\left(j-k\right)+CDIF_{PREDI;a;b;l}\left(j\right),$$

where the correction term $CDIF_{PREDI;b}$ is in Equation (32),

(32) $$CDIF_{PREDI;a;b;l}\left(j\right)≔CT_{PREDI;a;b}\left(\sum_{k=1}^{n}a_{k}f\left(k-i\right)-\sum_{k=1}^{n}a_{k}\;f_{l}\left(j-k\right)\right),$$

with the multiplier $CT_{PREDI;b}$ as:

(33) $$CT_{PREDI;a;b}≔\sum_{k=-m}^{0}b_{k}.$$

We choose from the *k* previous days the Day l for which the absolute value of the correction term $CDIF_{PREDI;b;l}$ is minimal and take the values $f_{l}$ from that day. Finally, Equation (31) is used to estimate the energy based on the next time slot, specifically for LINE-P (Case-I), and Equations (32) and (33) are the substitution factors of Equation (31).

**Time Complexity of LINE-P Case-I:** Typically, LINE-P case-I is dependent to the two parameters length of the kernel vector (*m*, *n*) and the number of previous days (*k*). The running time complexity of the correction term Equation (32) is *T*(*n*) = 2*nk*. Thus, the total running time complexity of Equation (31) for a single value estimation is *T*(*n*) = 2(*nk* + *m*) + 1. The Big-O notation of the LINE-P Case-I is *O*(*n*).

### 4.2. CASE-II

Generally, most prediction models predict energy based on the previous days, but here we propose a model which works with only n previous samples from the same day. For instance, if we suppose we do not have the samples from the previous days and have only few previous samples of the same day, in that case we can use those samples from the past to determine the current value of the function *t*. We can use the sampling operators (Equation (26)) with one-sided kernels where $a_{k}=0$ for k≤0, i.e.,

(34) $$(S_{PREDII;a}f)\left(j\right)≔\overset{m}{\underset{k=1}{\sum }}a_{k}f\left(j-k\right).$$

Here, Equation (34) is used for LINE-P (Case-II).

**Time Complexity of LINE-P Case-II:** LINE-P case-II is dependent to one parameter i.e., *m*. The running time complexity of LINE-P case-II is *T*(*n*) = *n*. Its notation in Big-O is *O*(*n*).

### 4.3. CASE-III

Specifically, in this case, if we have samples similar to in Case-I, the parameter vector *b* defines a symmetric kernel with the corresponding sampling operator (Equation (26)), yielding Equation (35).

(35) $$(S_{PREDIII;b}f)\left(j\right)≔\sum_{k=1}^{m}b_{k}f\left(j-k\right)+\sum_{k=-m}^{0}b_{k}\;f_{l}\left(j-k\right)+CDIF_{PREDIII;b;l}\left(j\right),$$

where the correction term $CDIF_{PREDIII;b:l}$ is in Equation (36),

(36) $$CDIF_{PREDIII;b;l}\left(j\right)≔CT_{PREDIII;b\;}\left(\sum_{k=1}^{m}b_{k}f\left(j-k\right)-\sum_{k=1}^{m}a_{k}\;f_{l}\left(j-k\right)\right),$$

with the multiplier $CT_{PREDIII;b}$

(37) $$CT_{PREDIII;b}≔\frac{\sum_{k=-m}^{0}b_{k}}{\sum_{k=1}^{m}b_{k}}.$$

We choose from the *k* previous days the day l for which the absolute value of the correction term $CDIF_{PREDIII;b;l}$ is minimal and take the values $f_{l}$ from that day.

Here, Equation (35) is used for LINE-P (Case-III).

**Complexity of LINE-P Case-III:** LINE-P Case-III is dependent on two parameters, i.e., the length of the kernel vector (*m*) and number of previous days (*k*). Considering the correction term (Equation (36)) and its time complexity which is *T*(*n*) = 2*mk*, now the total running time complexity of Equation (36) is *T*(*n*) = *m*(2*k* + 1) + 1. Its notation in Big-O is *O*(*m*).

## 5. Performance Comparison of LINE-P Model with the State-of-the-Art on Real Solar-Based Data Profiles

We evaluate the performance of the proposed LINE-P model (all three cases) based on solar profiles (datasets) in comparison with the state-of-the-art models by means of: (i) graphical representations along with real datasets; and (ii) calculating two types of errors.

### 5.1. Graphical Comparison of the Models for Solar Energy

In this section, we present the comparative analysis of the simulation results of all above-mentioned solar models, including LINE-P. They are examined on 22- and 60-min interval data corresponding to a medium case of 61 slots, and a longer case of 24 slots in 24 h, respectively. We show their graphical behavior in comparison with the real profiles (datasets) available in [9,10].

**Figure 2:**
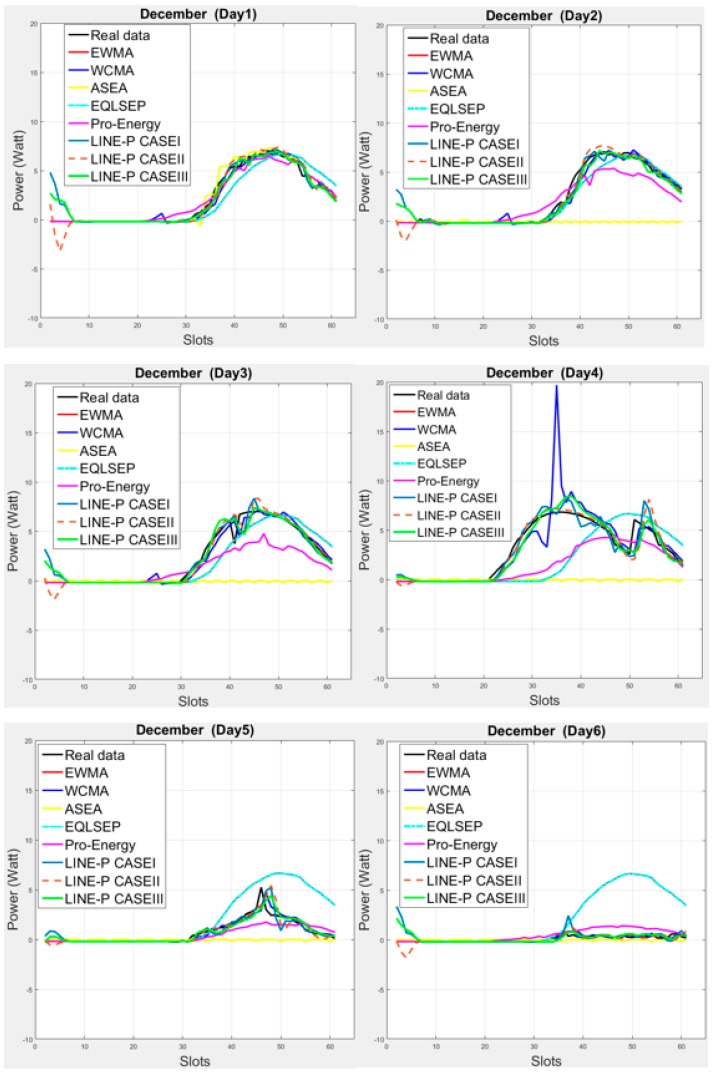
Graphical comparison of the obtained predictions for all energy prediction models (including the proposed LINE-P cases) for medium interval-based (61 slots) solar energy in December. Solar energy variations are troublesome for some of the models (e.g., EWMA and QL-SEP models on the 5th and 6th days, and ASEA on the 2nd day). On the other hand, ASEA is able to yield suitable predictions for longer intervals. In any weather situation, all three cases of LINE-P provide predictions very close to the real dataset.

Figure 2 illustrates the medium interval, considering 22-min interval data. As can be seen in the subplots, solar energy varies quite a lot, as shown here for the month of December. However, most proposed energy prediction models rely on the smaller number of slots (longer interval), as shown in the state-of-the-art and in Figure 2. The first four days appear quite consistent, but the next two days yields low energy production; such variations make that some of the models does not work properly in this situation. For example, for the fifth and sixth days, the predictions coming from the EWMA and QL-SEP models are quite off the real data. Another example is that ASEA collapses from the second day because it is not meant for medium and shorter slots. On the other hand, Figure 1 shows that ASEA is able to yield suitable predictions for longer intervals. As can also be observed, in any weather situation, all three cases of LINE-P provide predictions very close to the real dataset.

**Figure 3:**
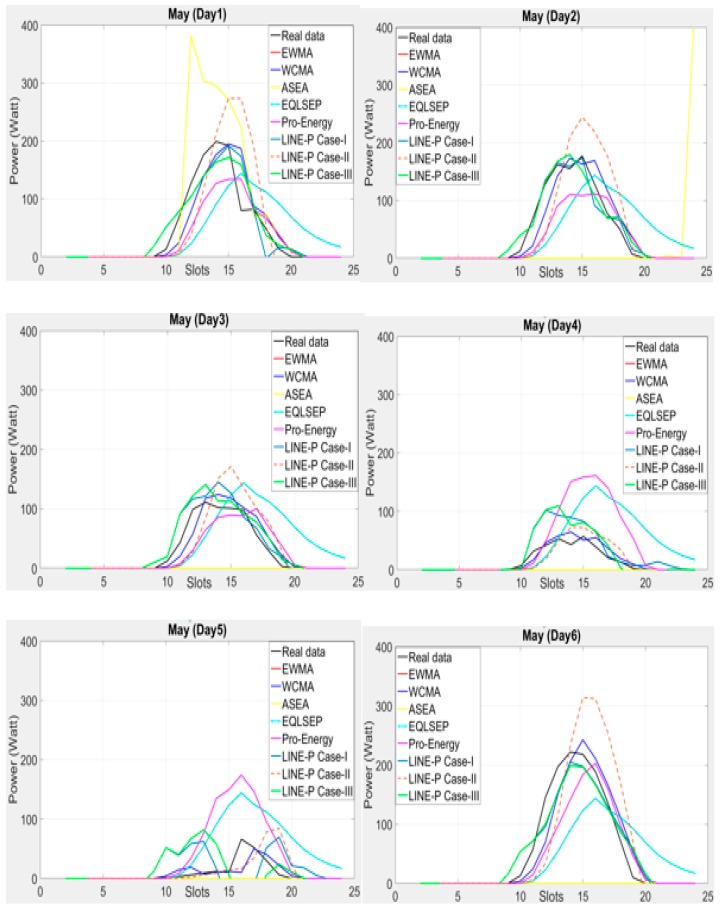
Graphical comparison of the obtained predictions for all models (including the proposed LINE-P cases) for longer interval-based (24 slots) solar energy in May. Here, even ASEA is not always able to deal with energy variations; in contrast, LINE-P Case-I and Case-III provide more realistic and accurate values.

As illustrated above the authors deploy real datasets [10] in all above models for the graphical comparison and use longer interval (24 slots) in a day. However, as Figure 3 shows, some of the models yield worst predictions such as EWMA, WCMA, Pro-Energy and QL-SEP. In addition, ASEA is also not an appropriate for this kind of datasets situation. On the contrary, LINE-P Case-I and Case-III provide more realistic and accurate values than the other models. Furthermore, among the three cases of LINE-P, Case-III performs better than Case-I and Case-II. In addition, note that, for certain days, Case-II yields over predictions.

In a nutshell, Figure 2 and Figure 3 clearly show that the proposed LINE-P model (all cases) are slots independent (adjustable based on the profiles) both for medium and short data intervals, as well as more reliable than the other models.

In the next section, the above graphical analysis is complemented by a mathematical error comparison in terms of mean square error and mean absolute error.

### 5.2. Error Comparison of the Models for Solar Energy

Mean square error (MSE) and mean absolute error (MAE) have been consider for comparing the error of each of model. To find the error in each model, we have used solar-based (SDG&E) [9] dataset (see Figure 3). We considered a medium interval (61 slots) in 24 h. As can be seen in Table 1, LINE-P (all cases) have the lowest error as compared to the other models. In addition, it is clearly visible that LINE-P Case-I and Case-III have lower MAE in all the days, as shown in Table 2.

## 6. Performance Comparison of LINE-P Model with Pro-Energy Models on Real Wind-Based Data Profiles

**Figure 4:**
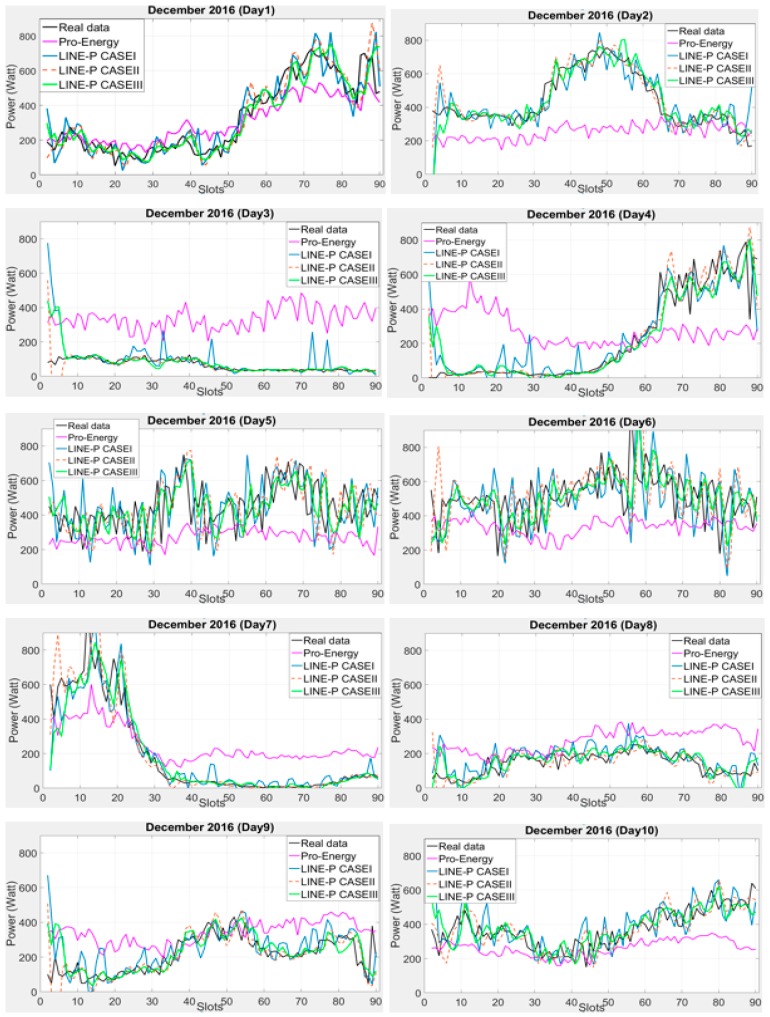
Graphical comparison of the obtained predictions for LINE-P (all cases) and Pro-Energy for shorter interval-based (90 slots) wind energy in December for dataset [9]. Generally speaking, LINE-P yields more accurate estimates than Pro-Energy.

**Figure 5:**
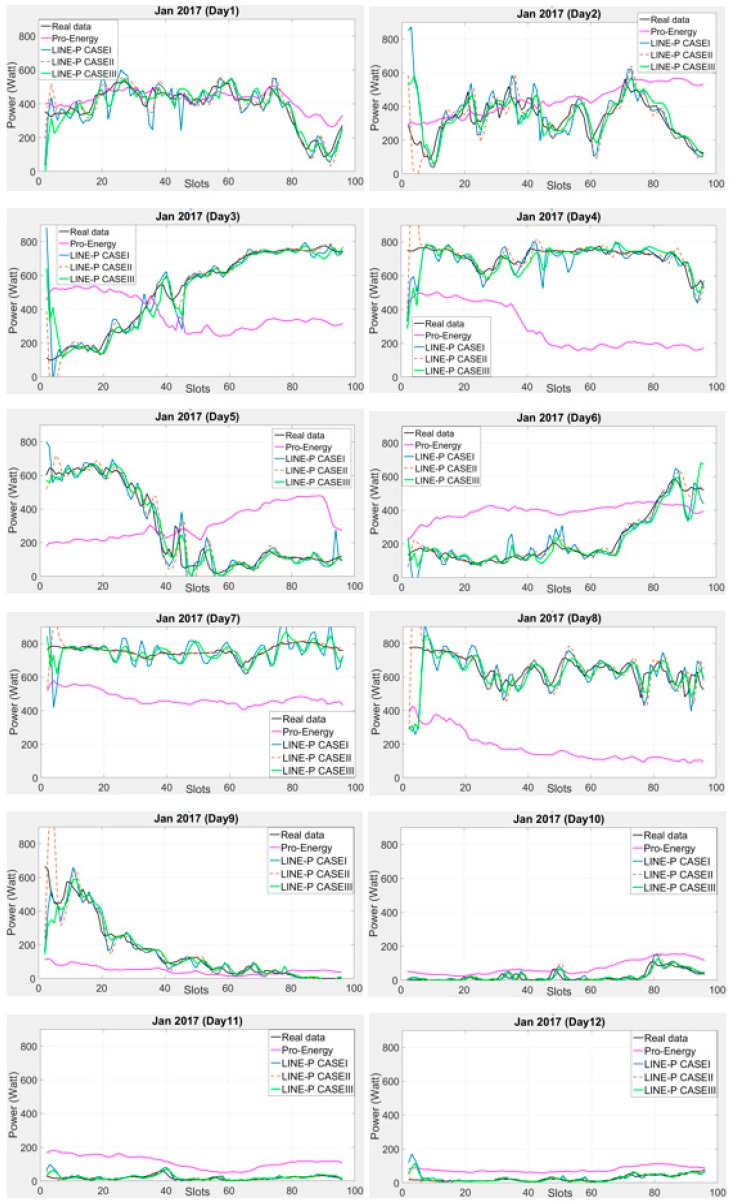
Graphical comparison of the obtained predictions for LINE-P (all cases) and Pro-Energy for shorter interval-based (96 slots) wind energy in December (12 days) and dataset [11]. Generally speaking, the estimates provided by LINE-P are more accurate than those of Pro-Energy.

Pro-Energy is suitable for both types of energy harvester (solar and wind) or multi-source harvesters [14,29]. Similarly, we designed LINE-P (all cases) keeping in mind dual-source EH (solar and wind). Furthermore, for the performance evaluation in terms of accuracy and robustness of the model, we have examined the proposed LINE-P with two different profile lengths (time slots) and conducted various experiments. We found very low error in LINE-P (all cases), as shown in Figure 4 and Figure 5. The previous section compared the performance of all models for solar energy; in what follows, we compare the performance of LINE-P and Pro-Energy for wind energy.

### 6.1. Graphical Representation of LINE-P and Pro-Energy Models for Wind Energy

The performance of the proposed LINE-P and of the existing Pro-Energy models have been examined for wind energy harvesting on a short 14.25-min data time interval of 90 slots in 24 h. Figure 4 shows their graphical behavior against the real profiles (i.e., US Department of energy [9] and National Laboratory of Research [10]). Figure 4 shows that for a 10-days dataset (real data), LINE-P (all cases) yields better results than Pro-Energy in most cases. Moreover, we also used a 15-min shorter data time interval of 96 slots in 24 h from another dataset [11]; the results shown in Figure 5 confirm that generally speaking, LINE-P performs better and more precisely than Pro-Energy. For example, the prediction yielded by Pro-Energy model in both Figure 4 and Figure 5 are over/under estimated for certain days. On the other hand, LINE-P (all cases), especially LINE-P Case-III, yields more vigorous, less complexity, compatible and accurate predictions. 

### 6.2. Error Comparison of the Models for Wind Energy

We also use MSE and MAE to compare the prediction errors of Pro-energy and LINE-P (all cases). In this case, we use datasets [11] to evaluate the prediction error. The results shown in Table 3 indicate that in general the prediction errors of LINE-P (all cases) are lower than that of Pro-Energy. From the results shown in Table 3, it is concluded that LINE-P (all cases) prediction values are very close to real data; especially Case-III is very effective and accurate.

### 6.3. Comparison of the Time Complexities

Table 4 shows the time complexity and Big-O notation for all prediction models. ASEA and EWMA have constant complexity (*O*(2)), whereas WCMA and Pro-Energy have quadratic complexities (*O*(*n*^2^) and *O*((*k* + 1)^2^), respectively). QL-SEP and LINE-P (all cases) have linear complexity (*O*(*n*) and *O*(*m*)).

Considering both the prediction performance of all models and their respective complexities, it can be said that the proposed LINE-P approach offers the best trade-off, i.e., equivalent or better prediction accuracy than the best existing models at a lower complexity. This means that LINE-P is a good candidate for embedded implementation on resource-constrained platforms such as WSN nodes/coordinators where CPU usage and energy consumption are critical.

### 6.4. Comparison of Space (Memory) Requirements

The proposed LINE-P model performs well as compared to the other models in terms of prediction error, and at the same time has small memory requirements. A higher number of slots *N* means memory overhead for a given predictor. For instance, assuming *N* = 48 and *D* (previous days) = 20, WCMA requires almost 4 kB of memory to store the matrix of *N*·*D* for an energy prediction [29]. On the contrary, LINE-P (Case-I) and (Case-III) use only require *N* = 13 and *D* = 4. Similarly, LINE-P (Case-II) only require *N* = 8 and *D* = 1. Thus, LINE-P models’ memory overheads are approximately 90% and 70% lower than for WCMA and Pro-Energy models, respectively.

## 7. Conclusions and Perspectives

We presented LINE-P (three cases-based) prediction model for dual-source (solar and wind energy harvesting) which is suitable for many possible data time intervals, e.g., shorter, medium and longer, as opposed to previous models that are only recommended for a particular data time interval (resulting in degraded predictions when slightly different conditions occur).

The proposed LINE-P (Case-I) predicts the energy based on the previous and current days. LINE-P (Case-II) predicts the energy according to the current days in case of missing data. LINE-P (Case-III) is a simplified version of LINE-P (Case-I): instead of the one-sided kernel, we use a part of the main symmetric kernel for measuring the closeness and error correction. Furthermore, LINE-P model allows adjusting or resizing of the kernels, making it compatible with solar powered WSNs. On the contrary, most of the solar-based prediction models exploit a fixed weighting parameter factor (*α*), which is incompatible with the solar harvesters due to their different parameter characteristics.

In addition, LINE-P’s principle means that it is associated with low computational and reduced memory overheads, making it suitable for implementation on WSN nodes/coordinators.

Several datasets have been considered to evaluate the prediction performance and error of the models. We found that LINE-P model provides low errors, for either solar or wind energy sources. In terms of MSE and MAE, the predictions are approximately 98% accurate for the LINE-P model Case-III for solar energy, and around 96% accurate for wind-based prediction.

As future work, we plan to extend LINE-P with adaptive features and compare its performance against those of UD-WCMA (adaptive tuning of the weighting factor) [4] and Pro-Energy-VLT (adaptive timeslots granularity) [5].

We also plan to integrate the proposed LINE-P model with our recent work on transient computing for WSNs, e.g., to dynamically control the execution patterns of the nodes depending on the available energy. In particular, we will develop an adaptive prediction model which will use the appropriate kernels according to the energy profiles; if the uncertainty thereof is high, then the model will use the non-sensitive kernels; on the other hand, if the energy profiles are smooth, then sensitive kernel will be used for higher prediction accuracy.

## Figures and Tables

**Table 1:** MSE of the LINE-P (all cases) and other prediction models for solar energy.

Prediction Model	1st Day MSE	2nd Day MSE	3rd Day MSE	4th Day MSE	Average MSE
EWMA	0.0169	0.0831	0.0546	0.0757	0.05757
WCMA	0.0029	0.0074	0.0215	0.0102	0.0105
ASEA	0.0081	0.4998	0.6539	0.6974	0.04648
QL-SEP	0.0169	0.0831	0.0546	0.0757	0.0575
Pro-Energy	0.0046	0.0395	0.0189	0.0299	0.02322
LINE-P (Case-I)	0.0032	0.0102	0.0388	0.0144	0.01665
LINE-P (Case-II)	0.0040	0.0125	0.0461	0.0181	0.020175
LINE-P(Case-III)	0.0038	0.0074	0.0296	0.0105	0.012825

**Table 2:** MAE of the LINE-P (all cases) and other prediction models for solar energy.

Prediction Model	1st Day MAE	2nd Day MAE	3rd Day MAE	4th Day MAE	Average MAE
EWMA	0.0820	0.2060	0.1588	0.2109	0.16442
WCMA	0.0388	0.0522	0.0863	0.0681	0.06135
ASEA	0.0472	0.5865	0.6379	0.6938	0.49135
QL-SEP	0.0820	0.2060	0.1588	0.2109	0.16442
Pro-Energy	0.0459	0.1493	0.0916	0.1319	0.104675
LINE-P (Case-I)	0.0426	0.064	0.1170	0.0743	0.074675
LINE-P (Case-II)	0.0407	0.0714	0.1279	0.0891	0.082275
LINE-P(Case-III)	0.0459	0.0574	0.0967	0.0682	0.06705

**Table 3:** Average MSE and MAE over 10 days for LINE-P (all cases) and Pro-Energy.

Prediction Models	10 Days MSE	10 Days MAE
Pro-Energy	0.777	0.238
LINE-P (Case-I)	0.028	0.038
LINE-P (Case-II)	0.021	0.031
LINE-P(Case-III)	0.018	0.032

**Table 4:** Time Complexity of the LINE-P (all cases) and the other prediction models. Note: In some models, we consider *m* and *k* times rather than *n* times.

Prediction Models	Time Complexity *T*(*n*)	Big-O Notation *O*(*n*)
EWMA	*T*(*n*) = 2	*O*(2)
ASEA	*T*(*n*) = 2	*O*(2)
WCMA	*T*(*n*) = *k*(*n*^2^ + 1)	*O*(*n*^2^)
Pro-Energy	*T*(*n*) = (*k* + 1)^2^*n*	*O*((*k* + 1)^2^)
QL-SEP	*T*(*n*) = (4*n* + 2)q	*O*(*n*)
LINE-P Case-I	*T*(*n*) = 2(*nk* + *m*) + 1	*O*(*n*)
LINE-P Case-II	*T*(*n*) = *n*	*O*(*n*)
LINE-P Case-III	*T*(*n*) = *m*(2*k* + 1) + 1	*O*(*m*)
